# Effects of calcitriol on oxidative burst, phagocytic function, and leukocyte cytokine production in shelter dogs

**DOI:** 10.1186/s40575-020-00090-y

**Published:** 2020-08-14

**Authors:** Jared A. Jaffey, Mariah Bessette, Zenan Tao, Nancy Bradley-Siemens, Melissa Thompson

**Affiliations:** 1 Department of Specialty Medicine, College of Veterinary Medicine, Midwestern University, 19555 N 59th Ave, Glendale, AZ 85308 USA; 2 Department of Pathology and Population Medicine, College of Veterinary Medicine, Midwestern University, 19555 N 59th Ave, Glendale, AZ 85308 USA; 3 Arizona Humane Society, 9226 N 13th Ave, Phoenix, AZ 85021 USA

**Keywords:** Vitamin D, 1,25(oh)_2_D, Respiratory burst, Phagocytosis, Innate immunity

## Abstract

**Background:**

The active metabolite of vitamin D, calcitriol, has been shown across many different species to augment innate immune responses and dampen aberrant proinflammatory cytokine production. Community acquired infections are common in shelters and consume limited shelter resources, impact adoption rates, and can result in unnecessary euthanasia. Prophylactic oral vitamin D supplementation decreases the incidence and severity of upper and lower respiratory tract infections in humans. Before a clinical trial investigating the clinical benefit of oral vitamin D supplementation in shelter dogs can be pursued, an in vitro study evaluating the immunomodulatory effects of calcitriol in blood from shelter dogs is warranted. Therefore, the objective of this study was to determine if incubation of whole blood obtained from apparently healthy dogs housed in a shelter for ≥7 days with calcitriol would alter granulocyte/monocyte (GM) oxidative burst and phagocytic function as well as pathogen-associated molecular pattern (PAMP)-stimulated leukocyte production of tumor necrosis factor (TNF)-α, and interleukin (IL)-6, and IL-10.

**Results:**

Ten dogs housed in a shelter for ≥7 days were enrolled in a prospective cohort study. Whole blood from these dogs was incubated with calcitriol (10^− 7^ M) or diluent (control) for 24 h. Subsequent to this incubation, phagocytosis of opsonized-*Escherichia coli* (*E. coli*) and *E. coli*-induced oxidative burst were evaluated via flow cytometry. In addition, leukocyte production of TNF-α, IL-6, and IL-10 were measured using a canine-specific multiplex bead assay. Calcitriol significantly decreased leukocyte TNF-α production (*p* = 0.009) and increased IL-10 production (*p* = 0.002). Tumor necrosis factor-α-to-IL-10 ratio was significantly decreased with calcitriol (*p* = 0.017), while IL-6 production as well as GM oxidative burst and phagocytic function were not significantly affected.

**Conclusions:**

These data indicate that calcitriol attenuates proinflammatory immune responses without affecting GM oxidative burst or phagocytic function in vitro in whole blood obtained from apparently healthy shelter dogs.

## Plain English summary

Vitamin D is an important hormone in musculoskeletal homeostasis but also has an integral role in the immune system. Several studies across many different species highlight that vitamin D enhances the immune response against infections. In fact, oral vitamin D supplementation has been shown to be well-tolerated and decreases the incidence of acute respiratory tract infections in people. Clinical trials in dogs that evaluate the benefit of oral vitamin D supplementation in the prevention of infections are lacking. Dogs housed in animal shelters commonly develop community acquired infections, which drains limited resources, decreases adoption rates, and can result in unnecessary euthanasia. Oral vitamin D supplementation in shelter dogs could have potential as an affordable and safe means to improve immune function and thus decrease the incidence of infections.

Before a clinical trial can be pursued, a proof of concept in vitro study is warranted to demonstrate that vitamin D has immunologic effects in shelter dogs. In this study we looked at the in vitro effect vitamin D had on the immune function of 10 apparently healthy dogs housed in a shelter. We found that the incubation of blood with vitamin D had significant anti-inflammatory effects without altering the ability of the immune cell to engulf (i.e., phagocytosis) or destroy (i.e., oxidative burst) the bacteria *Escherichia coli.*

These results indicate that vitamin D has immunologic effects in dogs housed in shelters and provides the needed rationale to pursue a future study that investigates immune function after oral vitamin D supplementation.

## Background

The active metabolite of vitamin D, calcitriol has been shown in many different species, including dogs, to augment innate immune responses [1–11]. Some of these protective effects include an increase in production of antimicrobial peptides, leukocyte reactive nitrogen/oxygen species and phagocytic capacities, and curtails deleterious proinflammatory cytokine responses [1–11]. These immunomodulatory functions highlight the importance of vitamin D to mucosal immunity and corroborates that hypovitaminosis D in people has been associated with increased susceptibility and severity of illness with inhaled respiratory pathogens [12–15].

Decreased 25-hydroxyvitamin (OH) D concentrations, the primary circulating metabolite of vitamin D, has been identified in many infectious diseases in dogs including leishmaniasis, babesiosis, spirocercosis, blastomycosis, and bacterial sepsis, which suggests that like people, vitamin D could have an important protective role in the canine innate immune response [16–20]. This theory is corroborated by early in vitro studies in healthy and critically ill dogs that demonstrated incubation of blood with calcitriol, decreased leukocyte production of the pro-inflammatory cytokine tumor-necrosis factor (TNF)-α in a concentration dependent manner [11, 21]. These in vitro results that have highlighted the protective immunomodulatory functions of vitamin D have translated to yield in vivo benefits with oral supplementation of vitamin D in people. A recent meta-analysis found that oral supplementation of vitamin D_2_ or vitamin D_3_ was well tolerated and decreased the incidence of acute respiratory tract infections in people [22]. Similar in vivo studies in dogs are lacking.

Dogs housed in animal shelters represent a unique population that could benefit from oral vitamin D supplementation. The biosynthesis of vitamin D in many species is initiated with exposure to ultraviolet light from the sun and warm ambient temperatures, wherein 7-dehydrocholesterol in the skin is transformed to pre-vitamin D_3_. Importantly, dogs are not able to synthesize vitamin D_3_ in their skin and thus, are dependent on diet to meet their vitamin D requirements [23]. It is reasonable to presume that confiscated and rescued dogs could have suboptimal vitamin D concentrations at the time of shelter admission in part because of inadequate nutrition. There is a high incidence of community acquired infections in dogs housed in animal shelters. These infections consume already limited resources, decrease adoption rates, and can even result in unnecessary euthanasia because of herd-health implications. Oral supplementation of vitamin D in dogs at the time of shelter admission could represent a cheap and safe therapy to decrease the incidence, severity of disease, or both of community acquired infections. Before a clinical trial can be pursued, an in vitro study investigating the immunomodulatory effects of vitamin D specifically in shelter dogs is warranted. The in vitro immunomodulatory effects of calcitriol has previously been investigated in healthy dogs [11]; however, dogs housed in animal shelters encounter various factors not accounted for in healthy dogs that could impact immune function including exposure to novel pathogens, stress associated with crowded environment, abrupt change of nutrition, and unknown pre-admission health care and husbandry.

Therefore, the objective of this study was to determine if incubation of whole blood obtained from apparently healthy dogs housed in a shelter for ≥7 days would alter granulocyte/monocyte (GM) oxidative burst and phagocytic function as well as pathogen-associated molecular pattern (PAMP)-stimulated leukocyte production of TNF-α, interleukin (IL)-6, and IL-10. We hypothesized that incubation of blood obtained from dogs housed in a shelter for ≥7 days would enhance GM oxidative burst and phagocytic function, while also decreasing PAMP-stimulated leukocyte production of pro-inflammatory cytokines (i.e., TNF-α and IL-6) with a concomitant increase in the anti-inflammatory cytokine, IL-10.

## Materials and methods

### Animals and selection criteria

The study was performed as a prospective, in vitro, cohort study. The study protocol was approved by the Midwestern University Animal Care and Use Committee (protocol # 2932). Ten randomly selected dogs from the Arizona Humane Society were eligible for enrollment in this study. Inclusion criteria consisted of being housed in the shelter for ≥7 days and tested negative for *Dirofilaria immitis* antigen, and antibodies to *Anaplasma phagocytophilum*, *Anaplasma platys, Ehrlichia canis*, *Ehrlichia ewingii,* and *Borrelia burgdorferi* C6 peptide using a commercial ELISA-based kit (SNAP 4DX, Plus Test kit, IDEXX Laboratories Inc., Westbrook, ME). Exclusion criteria included detection of ≥1 of the aforementioned pathogens. Furthermore, dogs were excluded if pregnant, lactating, had a surgical procedure performed, or had any illness since shelter admission. Furthermore, dogs were excluded if they had been administered medications, with the exception of routine parasitic prevention or fenbendazole.

### Calcitriol

Calcitriol (Sigma-Aldrich, St. Louis, MO) was dissolved in 75% ethanol (Sigma-Aldrich, St. Louis, MO) to make a stock solution of calcitriol at 24 nmol/mL and stored light protected at 4 °C as previously described [11].

### Blood sample collection and calcitriol treatment

A blood sample (8 mL) was collected from each dog via jugular venipuncture into tubes containing sodium heparin as an anticoagulant and processed within 1 h. Four mLs of blood was allocated into 2 separate 15 mL conical tubes for each dog. Blood samples in each conical tube were then diluted 1:2 with RPMI 1640 culture medium (Thermo Fisher Scientific, Carlsbad, CA) containing 200 U of penicillin/mL and 200 mg of streptomycin/mL. The blood-RPMI mixture was then incubated with calcitriol (final concentration, 10^− 7^ M) or ethanol negative control substance (final concentration, 6 × 10^− 2^ M) for 24 h at 37 °C in 5% CO_2_ in the dark as previously described [11].

### Leukocyte cytokine production

After incubation with calcitriol or control, blood-RPMI mixture was transferred to 96-well plates and stimulated with lipopolysaccharide (LPS) from *Escherichia coli* O127:B8 (final concentration, 100 ng/mL, Sigma Aldrich, St Louis, MO), lipoteichoic acid (LTA) from *Streptococcus faecalis* (final concentration, 1 μg/mL, Sigma Aldrich, St Louis, MO), or phosphate-buffered saline (PBS) control substance. Plates were incubated for 24 h at 37 °C in 5% CO_2_ in the dark. Following incubation, plates were centrifuged (400 X g for 7 min) at 21 °C as previously described [11]. The supernatant was collected and stored at − 80 °C for batch analysis. For analysis, samples were thawed, and then TNF-α, IL-6, and IL-10 were measured in supernatant with a canine cytokine-specific multiplex bead-based assay (Milliplex MAP canine cytokine-chemokine panel, EMD Millipore Corp, Billerica, MA) [11]. The median fluorescence intensity and cytokine concentration in each sample was measured in duplicate with appropriate controls and associated data analysis software (Milliplex Analyst version 5.1, EMD Millipore Corp, Billerica, MA). The lower limit of detection for TNF-α, IL-10 was 195 ρg/mL and IL-6 was 48.8 ρg/mL.

### Phagocytosis of *E. coli*

Phagocytic function of GM was determined with the PhagoTest commercial test kit (Orpegen Pharma, Heidelberg, Germany), validated for use in canines, according to manufacturer’s instructions. Briefly, 100 μL of blood-RPMI mixture post 24 h of incubation with calcitriol or control was then incubated in a 37 °C water bath for 10 min with 20 μL FITC-labeled, opsonized-*E. coli* bacteria or washing solution (negative control). The samples were placed on ice to arrest phagocytosis and 100 μL of quenching solution was added to remove the FITC-fluorescence of surface bound bacteria. The cells were washed, erythrocytes lysed, all cells were washed again, and 200 μL of DNA staining solution (R-phycoerythrin) was added to facilitate exclusion of aggregated artifacts of bacteria or cells.

### *E coli*-induced oxidative burst

Oxidative burst function in GM was determined with a PhagoBurst commercial test kit, validated for use in canines, according to manufacturer’s instructions (Orpegen Pharma, Heidelberg, Germany). Briefly, 100 μL of blood-RPMI mixture post 24 h of incubation with calcitriol or control was then incubated with 20 μL of opsonized-*E. coli* or control solution for 10 min at 37 °C in a water bath. Next, samples were incubated with 20 μL of dihydrorhodamine-123 as a fluorogenic substrate for oxygen intermediates for 10 min at 37 °C in a water bath. After cessation of this reaction, erythrocytes were lysed, the cells were washed, and 200 μL of DNA staining solution (R-phycoerythrin) was added to facilitate exclusion of aggregated artifacts of bacteria or cells.

### Flow cytometry

**Fig. 1:**
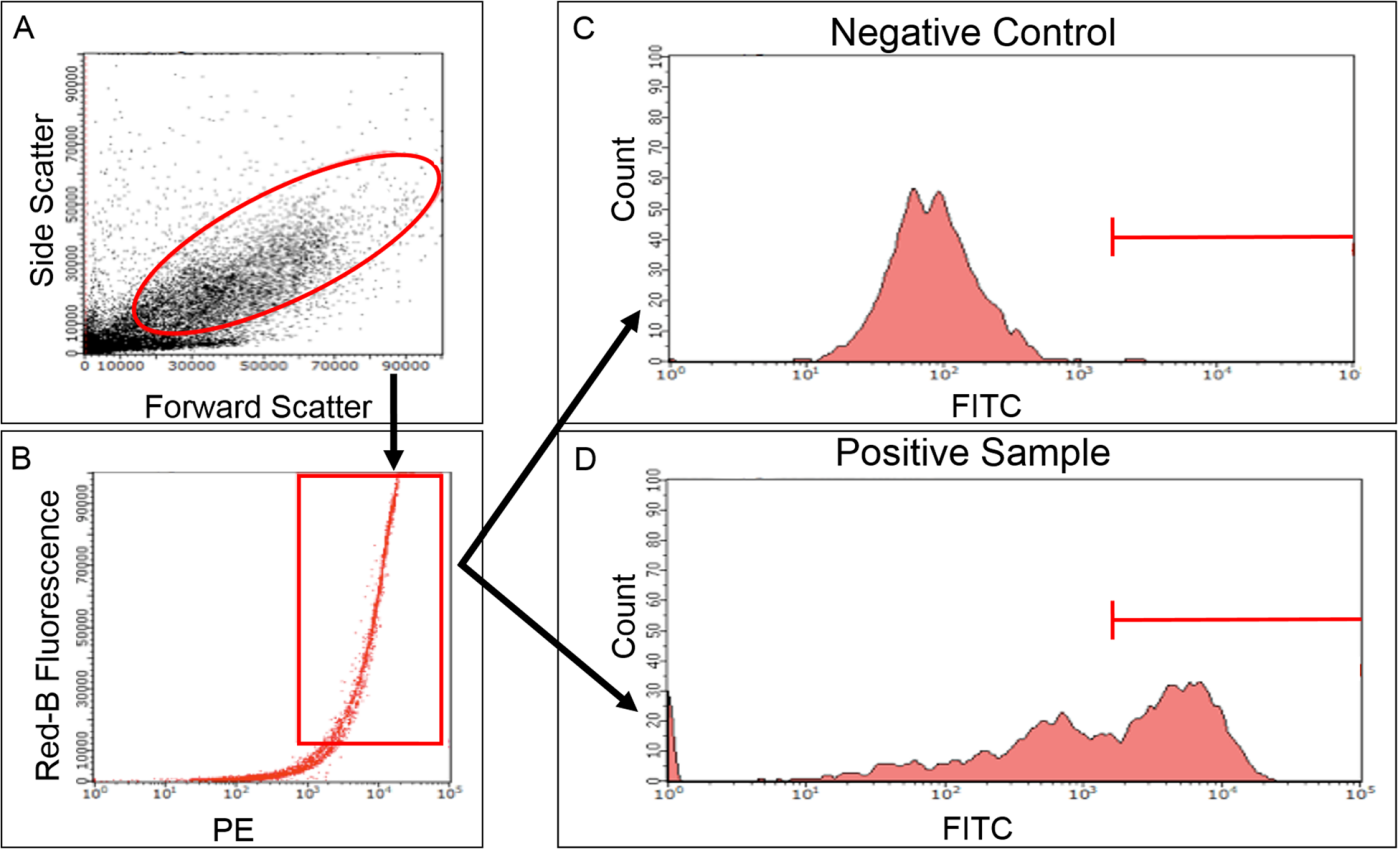
Gating scheme for flow cytometry of phagocytosis and oxidative burst. Granulocytes and monocytes/macrophages were gated on a forward versus side scatter plot (**a**), then R-phycoerythrin (PE)-labeled DNA stain was used to exclude aggregates of bacteria or dead cells and positive cells were identified and gated (**b**). This gate was then applied to appropriate histogram to identify FITC negative (**c**) and positive cells (**d**)

Flow cytometry was performed at the Midwestern University College of Veterinary Medicine Immunology Laboratory using a flow cytometer (Guava easyCyte HT, Luminex Corporation, Austin, TX) and associated data analysis software (GuavaSoft 3.2, Luminex Corporation, Austin, TX). A minimum of 20,000 events/sample were recorded. These events were then applied to a forward scatter versus side scatter plot to identify and gate GM populations concurrently (Fig. 1). For assessment of phagocytosis, data were recorded as the percentage of GM cells having internalized FITC-labeled *E. coli* as well as their mean fluorescent intensity (MFI), a method of quantifying the phagocytosed bacteria per cell. Data for assessment of oxidative burst were recorded as the percentage of GM cells having produced reactive oxygen metabolites and the MFI, the relative robustness of oxidative burst reaction produced per cell.

### Statistical analysis

Statistical analysis was performed by commercial software (SigmaStat, Systat Software Inc). Normality was determined using the Shapiro-Wilk test. Normally distributed data was presented as mean and standard deviation (SD), while data that was not normally distributed was presented as median and interquartile range (IQR). For between-treatment (i.e., calcitriol or control) comparisons (factor 1) for leukocyte production of each TNF-α, IL-6, and IL-10 after exposure with LPS, LTA, or PBS (factor 2), a two-way repeated measures analysis of variance (ANOVA) was performed with post hoc Bonferroni *t*-test making pairwise multiple comparisons. Granulocyte/monocyte oxidative burst and phagocytic function data were compared between treatment types (i.e., calcitriol or control) using paired *t*-tests. When the measured TNF-α, IL-6, and IL-10 concentrations fell below the lower limit of detection data were recorded at the lower limit of detection for statistical purposes. A *p*-value of < 0.05 was considered significant.

## Results

### Animal population

Sixteen dogs fulfilled the inclusion criteria. Six dogs were excluded because of various health derangements including one each for dehiscence of dental extraction sites, hit by car with lacerations and lameness, canine infectious respiratory disease complex, atopic dermatitis, facial wounds, and diarrhea. Ten dogs were enrolled. Breeds included Chihuahua (*n* = 4), Pit Bull Terrier (*n* = 3), and one each of American Bulldog, Australian Kelpie, and a mixed-breed dog. The average age was 3.4 years (SD, 1.8). There were 4 intact males, 3 spayed females, and 3 neutered males. The median weight and duration of time spent in shelter were 9.5 kg (IQR; 3.9–24.2) and 10 days (IQR; 9.8–12.0).

### Leukocyte cytokine responses

**Fig. 2:**
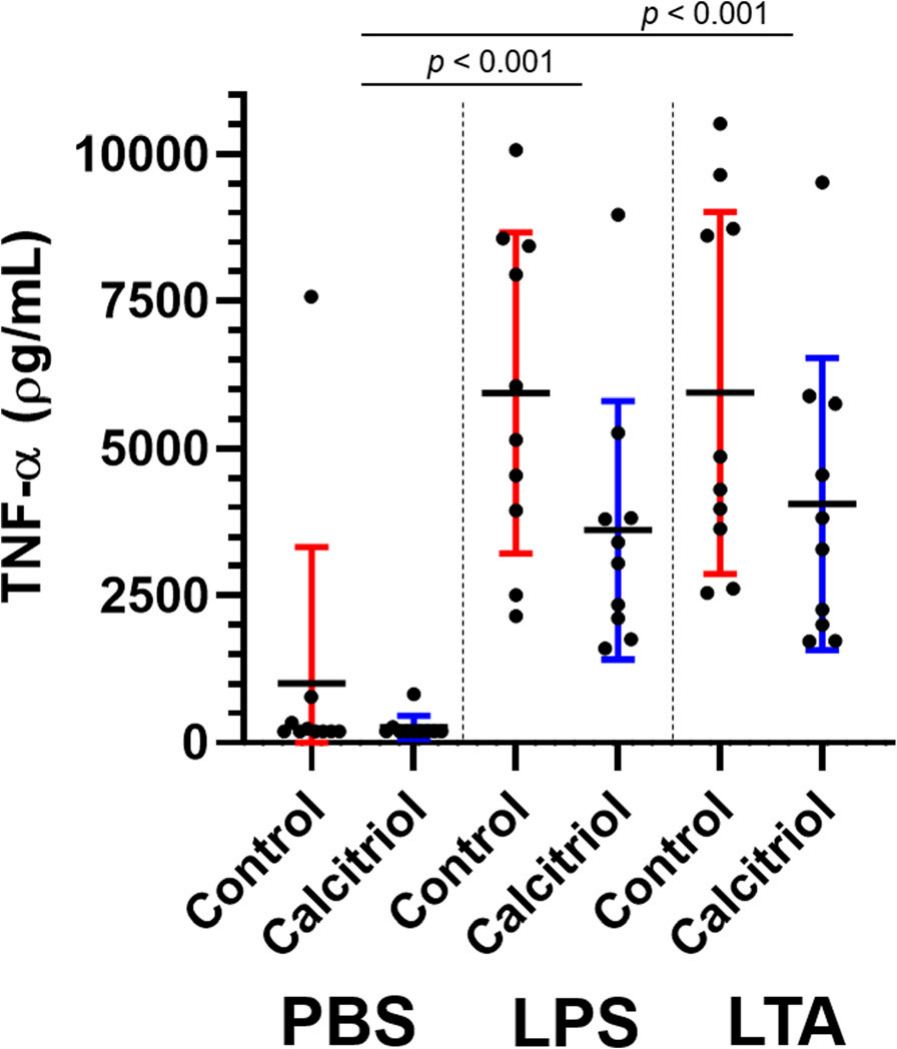
Two-way repeated measured analysis of variance comparing leukocyte production of tumor necrosis factor (TNF)-α between blood samples incubated in calcitriol (blue lines, *n* = 10) and control (red lines, *n* = 10) with subsequent exposure to phosphate buffered saline (PBS), lipopolysaccharide (LPS), or lipoteichoic acid (LTA). The black lines represent the mean, bars the SD, and the black circles are individual dog data. There were significant between-treatment (*p* = 0.009) and between-pathogen associated molecular pattern (PAMP)-exposure (*p* < 0.001) differences. There was not a treatment-by-PAMP exposure interaction (*p* = 0.08)

**Fig. 3:**
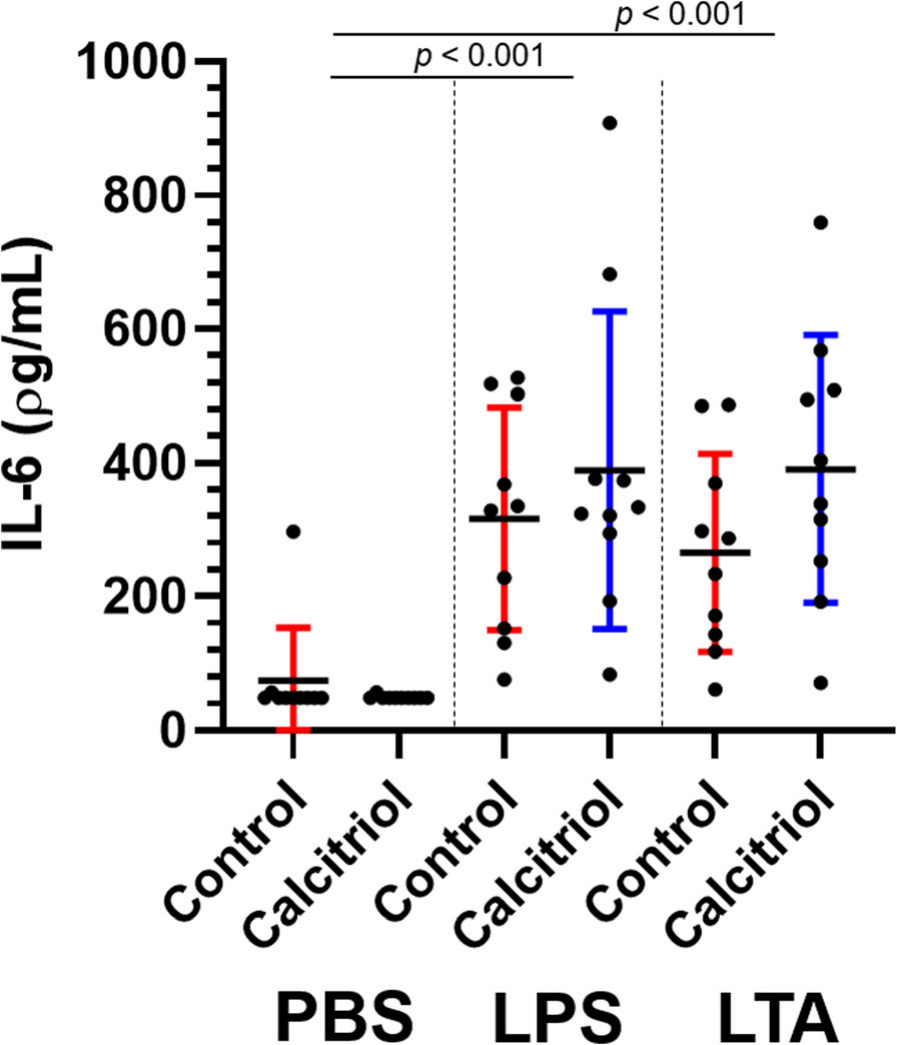
Two-way repeated measured analysis of variance comparing leukocyte production of interleukin (IL)-6 between blood samples incubated in calcitriol (blue lines, n = 10) and control (red lines, n = 10) with subsequent exposure to phosphate buffered saline (PBS), lipopolysaccharide (LPS), or lipoteichoic acid (LTA). The black lines represent the mean, bars the SD, and the black circles are individual dog data. There was a significant between-pathogen associated molecular pattern (PAMP)-exposure difference (*p* < 0.001), but not a between-treatment difference (*p* = 0.12). There was not a treatment-by-PAMP exposure interaction (*p* = 0.07)

**Fig. 4:**
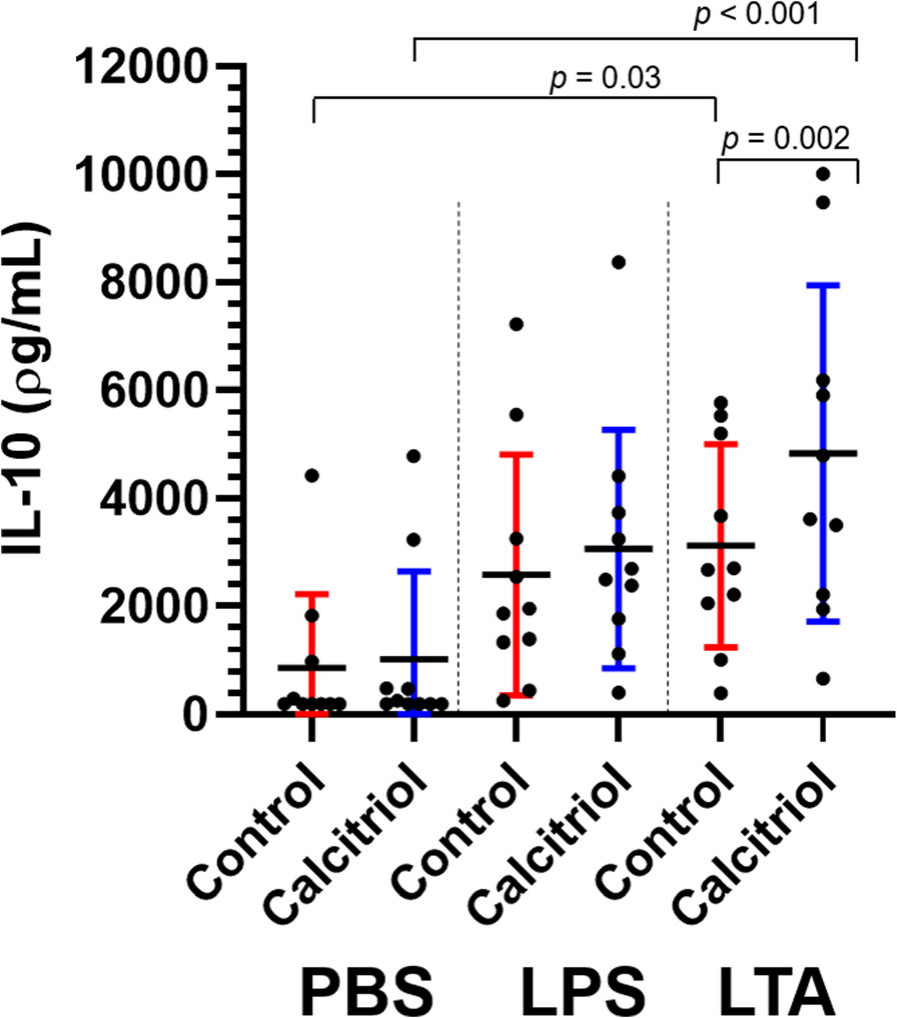
Two-way repeated measured analysis of variance comparing leukocyte production of interleukin (IL)-10 between blood samples incubated in calcitriol (blue lines, n = 10) and control (red lines, n = 10) with subsequent exposure to phosphate buffered saline (PBS), lipopolysaccharide (LPS), or lipoteichoic acid (LTA). The black lines represent the mean, bars the SD, and the black circles are individual dog data. There was a significant treatment-by-pathogen associated molecular pattern-exposure interaction for leukocyte production of IL-10 (*p* = 0.01)

There was a significant between-treatment difference in leukocyte production of TNF-α (*p* = 0.009) but not IL-6 (*p* = 0.12), irrespective of type of PAMP-exposure (i.e., LPS, LTA, PBS; Figs. 2, 3). Post hoc tests revealed that leukocyte production of TNF-α significantly decreased when cells were incubated with calcitriol compared to control (Fig. 2). A significant between-PAMP-exposure difference in leukocyte production of TNF-α (*p* < 0.001) and IL-6 (*p* < 0.001), irrespective of cell treatment (i.e., calcitriol or control) was identified (Figs. 2, 3). As expected, post hoc tests revealed that LPS- and LTA-exposed leukocytes produced significantly greater TNF-α and IL-6 than leukocytes exposed to PBS (*p* < 0.001; Figs. 2, 3). There was a significant treatment-by-PAMP-exposure interaction for leukocyte production of IL-10 (*p* = 0.01; Fig. 4). Therefore, between-treatment and between-PAMP-exposure differences for IL-10 could not be measured because of this interaction. Post hoc tests revealed that cells incubated with calcitriol produced significantly greater IL-10 compared with control when exposed with LTA (*p* = 0.002; Fig. 4). Further, these post hoc tests showed that cells incubated with calcitriol (*p* < 0.001) or control (*p* = 0.03) produced significantly greater IL-10 when exposed with LTA compared to PBS (Fig. 4).

### TNF-α-to-IL-10 ratio

**Fig. 5:**
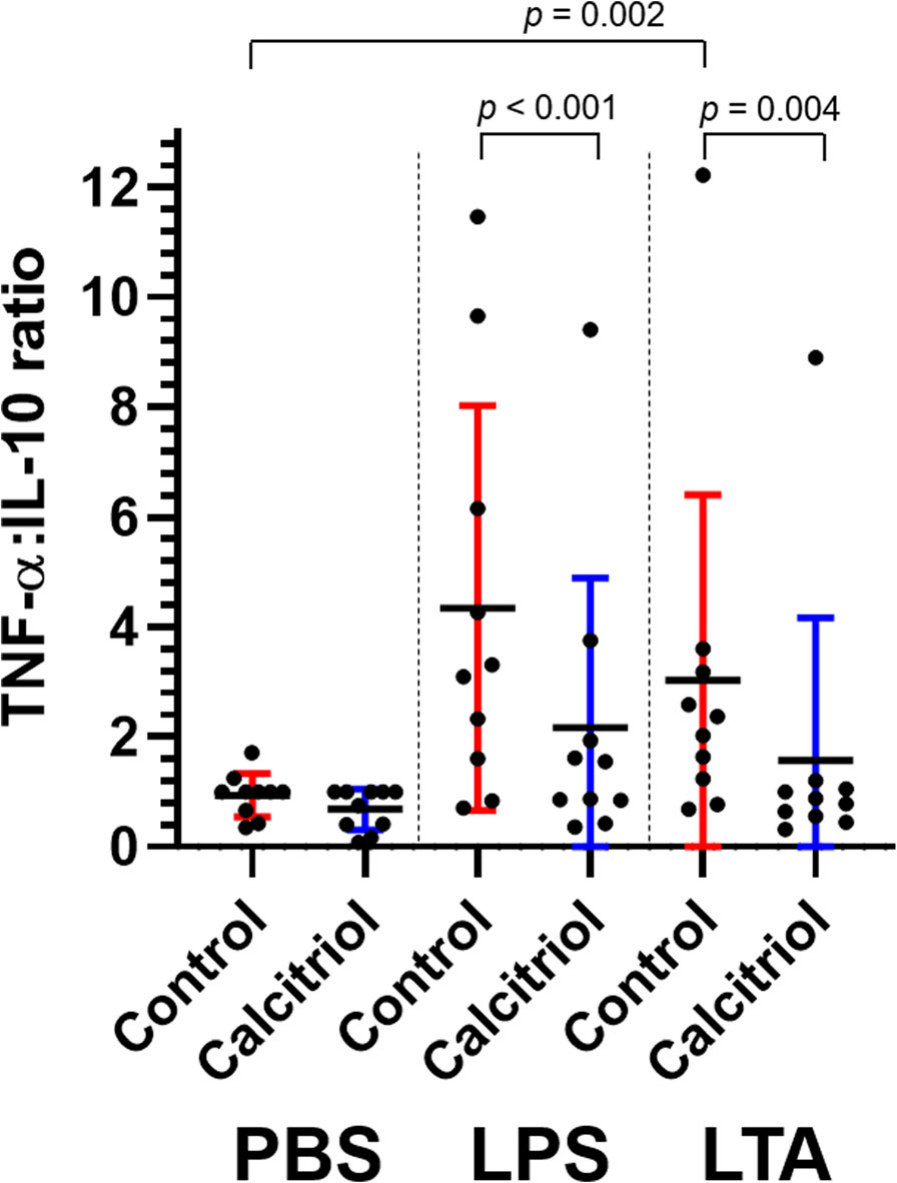
Two-way repeated measured analysis of variance comparing tumor necrosis factor (TNF)-α-to-interleukin (IL)-10 ratios between blood samples incubated in calcitriol (blue lines, n = 10) and control (red lines, n = 10) with subsequent exposure to phosphate buffered saline (PBS), lipopolysaccharide (LPS), or lipoteichoic acid (LTA). The black lines represent the mean, bars the SD, and the black circles are individual dog data. There was a significant treatment-by-pathogen associated molecular pattern-exposure interaction for TNF-α-to-IL-10 ratio (*p* = 0.017)

A significant treatment-by-PAMP exposure interaction was identified for TNF-α-to-IL-10 ratio precluding analysis of between-treatment and between-PAMP exposure differences (*p* = 0.017; Fig. 5). Post hoc tests revealed that incubation of cells with calcitriol resulted in a significant decrease in TNF-α-to-IL-10 ratio compared to control, when cells were exposed to LPS (*p* < 0.001) or LTA (*p* = 0.004; Fig.5). In addition, as expected, LPS-exposure in cells yielded a significantly greater TNF-α-to-IL-10 ratio than PBS-exposed cells incubated with control (*p* = 0.002; Fig. 5).

### Phagocytosis and oxidative burst

**Fig. 6:**
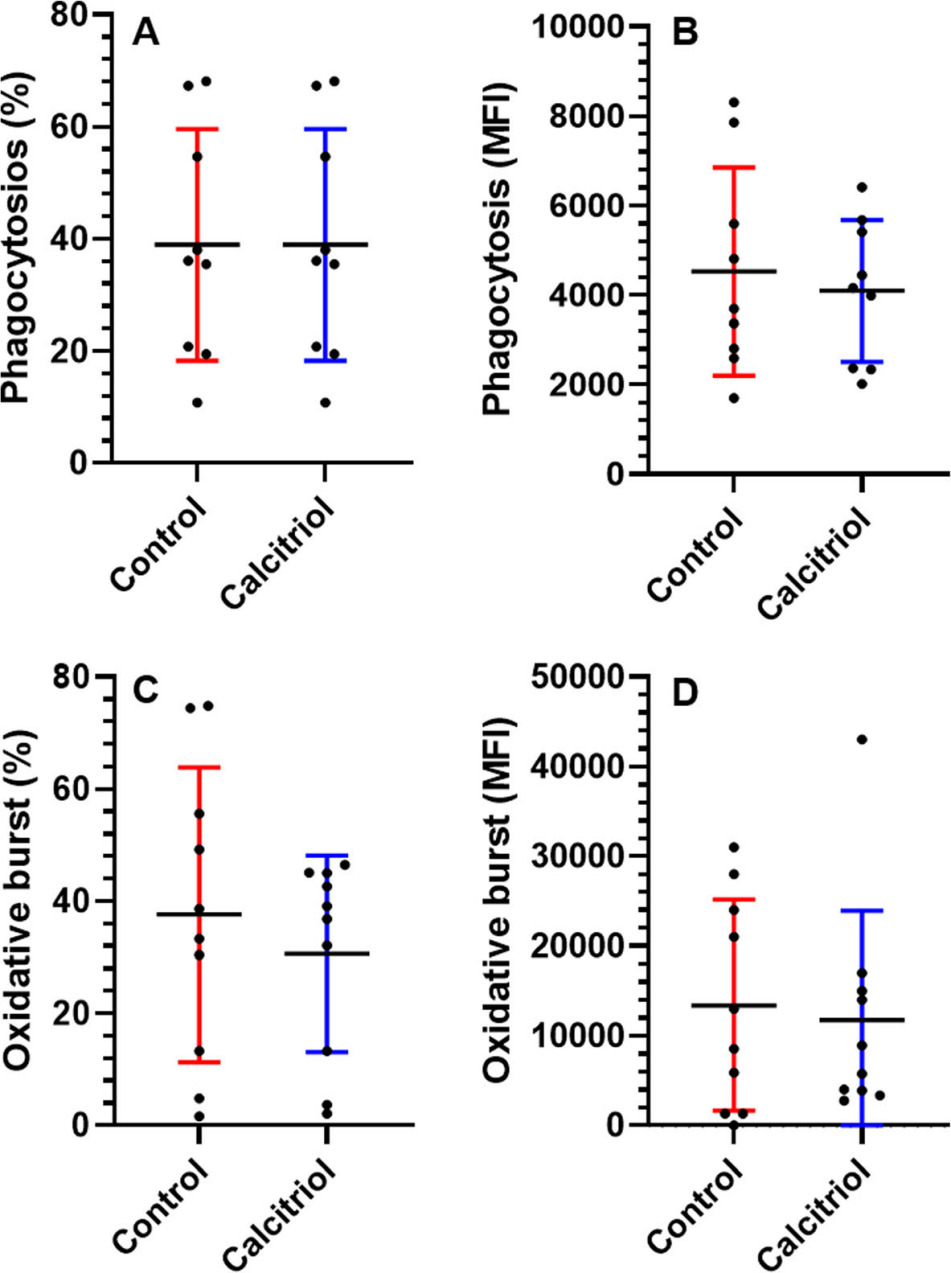
Two-tailed paired *t*-test comparing the percentage of granulocytes/monocytes (GM) that phagocytized opsonized-*E. coli* (A, *n* = 9) and performed *E. coli*-induced oxidative burst (C, n = 10) as well as the number of phagocytized opsonized-*E. coli* per cell (B, n = 10) and the intensity of *E. coli*-induced oxidative burst per cell (D, n = 10) in samples incubated with calcitriol (blue lines) or control (red lines). The black lines represent the mean, bars the SD, and the black circles are individual dog data

There was not a significant difference in the percentage of GM that phagocytized opsonized-*E. coli* or number of phagocytized opsonized-*E. coli* per cell between blood samples incubated with calcitriol or control (Fig. 6a,b). Technical difficulties precluded the inclusion of phagocytosis results from 1 dog. Likewise, a significant difference in the percentage of GM that had performed *E.coli*-induced oxidative burst as well as the *E.coli*-induced oxidative burst per cell was not identified (Fig. 6c,d).

## Discussion

In the present study, we evaluated the effects of calcitriol on leukocyte cytokine production and GM *E. coli-* induced-oxidative burst and phagocytosis of opsonized-*E. coli* in blood samples obtained from apparently healthy dogs housed in a shelter for ≥7 days. Incubation of blood with calcitriol resulted in a decreased leukocyte production of TNF-α, irrespective of the type of PAMP-exposure. Leukocytes incubated with calcitriol produced greater IL-10 with subsequent LTA-exposure. There was not a between-treatment (i.e., incubation with calcitriol or control) difference in leukocyte production of IL-6. The inflammatory milieu when evaluated in the context of TNF-α-to-IL-10 ratio, was shifted to an anti-inflammatory phenotype when blood was incubated with calcitriol when exposed to LPS and LTA. Calcitriol did not affect *E. coli*-induced oxidative burst or phagocytosis of opsonized-*E. coli*.

Incubation of blood with calcitriol resulted in a decrease in leukocyte production of TNF-α, irrespective of the type of PAMP-exposure. This finding supports our hypothesis and corroborates results from previous studies that demonstrated calcitriol decreased leukocyte production of TNF-α in blood obtained from healthy and critically-ill dogs [11, 21]. The molecular etiology for calcitriol induced modulation of TNF-α production in dogs is largely unknown. However, there are multiple proposed mechanisms in humans that are postulated to explain this process. Ligation of toll-like receptors (TLR) with their respective PAMP initiates a cascade of signaling pathways that result in the induction of proinflammatory cytokines (e.g., TNF-α, IL-6, IL-1). In humans and rodents, studies have illustrated that calcitriol decreases monocyte expression of TLR-2 and TLR-4 resulting in a relative decrease in the quantity of available receptors to propagate inflammation [24, 25]. A small in vitro pilot study in dogs did not find that calcitriol had a significant effect on GM TLR-4 expression [11]. Calcitriol also suppresses the activation and signaling of proinflammatory pathways including nuclear factor (NF)-kB and mitogen-activated protein kinase (MAPK) [26, 27]. Interestingly, calcitriol did not decrease leukocyte production of IL-6 in this study. The reason for this lack of response is unknown and could be related to the small study population. This proinflammatory cytokine is influenced by the same molecular pathways that mediate production of TNF-α (i.e., NFkB and MAPK). Additional studies with larger populations are needed to better understand the effect calcitriol has on leukocyte production of IL-6 in dogs.

During infection, the host innate immune response generated against pathogens can be unbalanced and potentially detrimental. Tumor necrosis factor-α has long been implicated in the development of immunopathology and tissue injury [28]. The deleterious effects of TNF-α are conserved across various species, including dogs. To the authors’ knowledge, the role of proinflammatory cytokines including TNF-α on morbidity and mortality of shelter and non-shelter dogs with spontaneous community-acquired infections has not been investigated. However, studies in humans illustrate that many community-acquired upper and lower respiratory infections are associated with exaggerated production of proinflammatory cytokines including TNF-α [29]. Moreover, studies have demonstrated that TNF-α activity is associated with the severity of disease and prolonged production of TNF-α exacerbates illness [30–32]. Future clinical trials are needed to investigate if prophylactic oral administration of vitamin D to shelter dogs dampens aberrant production of TNF-α.

The incubation of leukocytes with calcitriol significantly increased production of IL-10 when exposed to LTA, but not LPS. These results indicate that calcitriol could partially abrogate aberrant host inflammatory response when exposed to gram positive bacteria. The immunomodulatory effect that calcitriol has on PAMP-exposed leukocyte IL-10 production has a great deal of intra- and interspecies variability [33–36]. A previous small pilot study in dogs determined that calcitriol did not increase leukocyte production of IL-10 when exposed to LPS or LTA [11]. A subsequent series of in vitro studies showed that priming of cells (e.g., endotoxin or endogenous inflammatory mediators) before incubation with calcitriol could be one factor that is needed for an increase in leukocyte production of IL-10 with subsequent LPS-exposure [37]. The reason for a potential contrasting production capacity of IL-10 in cells incubated with calcitriol when exposed to LPS versus LTA as was seen in this study is unknown. In humans, calcitriol bound to its receptor directly increases expression of IL-10 independent of the pathways mediated by TLR/PAMP ligation [38].

Calcitriol significantly decreased the TNF-α-to-IL-10 ratio with post-incubation exposure of LPS and LTA. Tumor necrosis factor-α-to-IL-10 ratio is often used to investigate shifts in host inflammatory phenotype [39]. Our results indicate that pre-incubation of leukocytes obtained from shelter dogs with calcitriol dampens the proinflammatory response to post-incubation PAMP-stimulation. It is also important to consider that a decreased TNF-a-to-IL-10 ratio is not uniformly an immunologic advantage. This tolerogenic shift dampens potentially deleterious exaggerated inflammation, but this could also coincide with a compromised immune response to pathogens. Community-acquired infections, especially community acquired respiratory disease complex has a high prevalence in shelters [40]. The highly infectious nature of the complex represents a great burden on shelter resources. Prophylactic oral supplementation of vitamin D has been shown to decrease the incidence and severity of respiratory infections in humans [22, 41]. Future clinical trials are needed to investigate if prophylactic oral administration of vitamin D to shelter dogs’ decreases TNF-α-to-IL-10 ratio in vivo and if this translates to decreased incidence, morbidity, or both of community acquired infections.

In contrast to human monocytes/macrophages, incubation of cells with calcitriol did not significantly affect leukocyte phagocytic or oxidative burst functions in our apparently healthy shelter dogs [6, 10, 42–44]. Our study evaluated the effect of calcitriol on GM phagocytosis and oxidative burst associated with *E. coli*. Many of the aforementioned studies in humans specifically investigated the in vitro effect that calcitriol had on monocyte/macrophage immune responses to *Mycobacterium tuberculosis*. Pathogen recognition, phagocytosis, and subsequent pathogen killing is a selective process, requiring specific interactions between host immune cell receptors and pathogens. *Mycobacterium tuberculosis* interacts with various different TLRs including 2, 4, 8, and 9 found predominately with macrophages [45]. In contrast, *E. coli* interacts with most cells in the innate immune system via TLR-4 alone. While most human studies highlight a uniformly protective effect of calcitriol against *Mycobacterium tuberculosis*, there are conflicting reports on the benefit of calcitriol against *E. coli* [46, 47]. Therefore, it is possible that calcitriol has differential immune responses dependent on the type of pathogen encountered by the host. In addition, this study utilized a single concentration of calcitriol (i.e., 10^− 7^ M) and incubation time period (i.e., 24 h). Additional studies that utilize various calcitriol concentrations and incubation times are needed.

Our study had several limitations. We utilized whole blood cultures to evaluate various effects on immune responses. This global approach allowed us to investigate the broad and diverse effects of calcitriol in a manner more relatable to in vivo microenvironments and immune interactions. This study used *E. coli* as the model micro-organism in its in vitro evaluation of GM phagocytic and oxidative burst capacities. This approach limits the interpretation of these results to pathogens that ligate TLR-4 (i.e., a gram-negative bacterium like *E. coli*). These results could be representative of the canine innate immune response *to Bordetella bronchiseptica*, a common gram-negative CIRD pathogen that ligates TLR-4; however, they do not reflect the immune response to other bacterial and viral pathogens that utilize other immunologic pathways. Additional studies are needed to explore the in vitro effect of calcitriol with micro-organisms that utilize a broader array of leukocyte recognition pathways. In addition, the short-term incubation of whole blood with calcitriol may not replicate the in vivo immunomodulatory effects of oral supplementation with vitamin D in dogs. Eventual in vivo clinical trials are needed to further explore this. Importantly, the results from this in vitro study should not be extrapolated into in vivo clinical situations. We investigated the effect of calcitriol on specific immune functions. In other words, these tests were performed in a vacuum and the net effect could be different and potentially detrimental to clinical patients. Future in vivo studies are needed before vitamin D can be applied as a therapeutic intervention in dogs. Another limitation is that we enrolled only apparently healthy dogs. Calcitriol could have a different effect on shelter dogs that were systemically ill because they would be expected to have decreased serum 25(OH) D concentrations compared to healthy dogs [20]. Humans that are vitamin D deficient experience the most clinical benefit when supplemented with oral vitamin D [22].

## Conclusions

These data indicate that calcitriol is capable of shifting the phenotype of leukocytes obtained from shelter dogs from proinflammatory to anti-inflammatory. Calcitriol did not affect GM phagocytosis of opsonized-*E. coli* or *E. coli*-induced oxidative burst. Future in vitro and in vivo studies in healthy and sick shelter dogs are needed to better understand the potential therapeutic benefit of oral vitamin D supplementation.

## Data Availability

The datasets used and/or analyzed during the current study are available from the corresponding author on reasonable request.
